# Factors Contributing to Epidemic MRSA Clones Replacement in a Hospital Setting

**DOI:** 10.1371/journal.pone.0043153

**Published:** 2012-08-14

**Authors:** Rossella Baldan, Francesca Testa, Nicola I. Lorè, Alessandra Bragonzi, Paola Cichero, Cristina Ossi, Anna Biancardi, Paola Nizzero, Matteo Moro, Daniela M. Cirillo

**Affiliations:** 1 Emerging Bacterial Pathogens Unit, San Raffaele Scientific Institute, Milan, Italy; 2 Infection and Cystic Fibrosis Unit, San Raffaele Scientific Institute, Milan, Italy; 3 Laboraf, Diagnostic and Research San Raffaele, Milan, Italy; 4 Chief Medical Office, San Raffaele Scientific Institute, Milan, Italy; Rockefeller University, United States of America

## Abstract

The mechanisms governing the epidemiology dynamics and success determinants of a specific healthcare-associated methicillin-resistant *S. aureus* (HA-MRSA) clone in hospital settings are still unclear. Important epidemiological changes have occurred in Europe since 2000 that have been related to the appearance of the ST22-IV clone. Between 2006 and 2010, we observed the establishment of the ST22-IV clone displacing the predominant Italian clone, ST228-I, in a large Italian university hospital. To investigate the factors associated with a successful spread of epidemic MRSA clones we studied the biofilm production, the competitive behavior in co-culture, the capacity of invasion of the A549 cells, and the susceptibility to infection in a murine model of acute pneumonia of the two major HA-MRSA clones, ST22-IV and ST228-I. We showed that persistence of ST22-IV is associated with its increased biofilm production and capacity to inhibit the growth of ST228-I in co-culture. Compared to ST228-I, ST22-IV had a significantly higher capacity to invade the A549 cells and a higher virulence in a murine model of acute lung infection causing severe inflammation and determining death in all the mice within 60 hours. On the contrary, ST228-I was associated with mice survival and clearance of the infection. ST22-IV, compared with ST228-I, caused a higher number of persistent, long lasting bacteremia. These data suggest that ST22-IV could have exploited its capacity to i) increase its biofilm production over time, ii) maintain its growth kinetics in the presence of a competitor and iii) be particularly invasive and virulent both *in vitro* and *in vivo,* to replace other well-established MRSA clones, becoming the predominant European clone.

## Introduction

Infections caused by antibiotic-resistant strains of *Staphylococcus aureus* have risen to epidemic proportions globally [1]. The overall burden of disease caused by methicillin-resistant *S. aureus* (MRSA) strains has reached alarming rates in many countries in both healthcare and community settings [2]–[4].

MRSA, for its ability to become resistant to virtually all antimicrobial agents available, to survive in hostile environments and to adapt to new ecological niches poses a major challenge to Infection Control Programs. After successfully spreading to hospitals and healthcare settings worldwide (HA-MRSA, healthcare-associated MRSA), two decades ago MRSA emerged in the community (CA-MRSA, community-acquired MRSA), causing infection in healthy people without predisposing risk factors for HA-MRSA infection. CA- and HA-MRSA strains can be distinguished for their genetic background, phenotype and virulence factors. CA-MRSA strains are susceptible to most non β-lactams antibiotics, and contain smaller, more mobile SCC*mec* allotypes, SCC*mec* IV, V and VII, and virulence factors genes, such as those coding for the Panton Valentine leucocydin (PVL), which is responsible, in combination with other exotoxins, for their enhanced pathogenicity. Since 2003, MRSA has been isolated from livestock and humans exposed to infected animals in several countries, revealing the zoonotic potential of MRSA. Such MRSA has been dubbed livestock-associated methicillin-resistant *Staphylococcus aureus* (LA-MRSA) [5], [6]. Interactions between these different reservoirs have been reported, including nosocomial infections by CA-MRSA [7], [8] and LA-MRSA [9], [10].

Epidemiological studies using several molecular typing methods have shown that few highly epidemic MRSA (EMRSA) clones cause the majority of HA-MRSA infections worldwide. These EMRSA clones emerged upon acquisition of the SCC*mec* element by successful methicillin susceptible *S. aureus* clones, belonging to five phylogenetically distinct clonal complexes (CC): CC5 (sequence type ST5-I, -II, -IV, -VI), CC8 (ST247-I, ST239-III, ST8-IV), CC22 (ST22-IV), CC30 (ST36-II) and CC45 (ST45-II, -IV) [11], [12].

Since 2000, important epidemiological changes have occurred in Europe: the frequency of multidrug resistant (MDR) MRSA clones gradually decreased while the frequency of other epidemic clones still sensitive to non β-lactams, such as ST22-IV (EMRSA-15), increased [13]–[19].

A recent study, that traced the dynamic changes of HA-MRSA lineages in Italy, showed that over a 17-year period MDR-MRSA clones ST247-I/IA and ST239-III were replaced by ST228-I (South German), ST8-IV (EMRSA-2/−6) and ST22-IV [20]. Using pulsed-field gel electrophoresis and SCC*mec* typing, we characterized 575 clinically relevant, non-duplicated MRSA strains (499 index cases and 76 secondary cases due to nosocomial transmission), isolated from 2006 to 2010 at the San Raffaele hospital (hSR), a large private university hospital located in Milan. We identified three main epidemic HA-MRSA clones: ST22-IV (33,3% of isolates, considering only the index cases), ST228-I (26%) and ST8-IV (15,8%). During the 5-year study period, ST22-IV became the predominant clone in hSR, replacing the most prevalent Italian clone, ST228-I. Similar shifts have been observed in several European countries [15]–[18], [21].

The mechanisms involved in the selection of pandemic MRSA clones in nosocomial settings have not yet been clarified. In order to investigate the factors associated with the successful establishment and spread of an epidemic MRSA clone, as well as with the phenomenon of clone displacement in hospital environments, we studied both *in vitro* and *in vivo* virulence properties of the two major HA-MRSA clones, ST22-IV and ST228-I. In particular, we evaluated their ability to produce biofilm, their *in vitro* competitive behavior in co-culture and their capacity to invade the A549 cell line, as well as the virulence in a murine model of acute pneumonia.

## Results

### Biofilm Production

Selected MRSA strains belonging to both epidemic (ST228-I, ST22-IV, ST8-IV) and sporadic (ST5-II, ST239-III, ST1-IV, ST8-IV USA300) clones were tested for their capacity to produce biofilm *in vitro* (see Materials and Methods).

**Figure 1:**
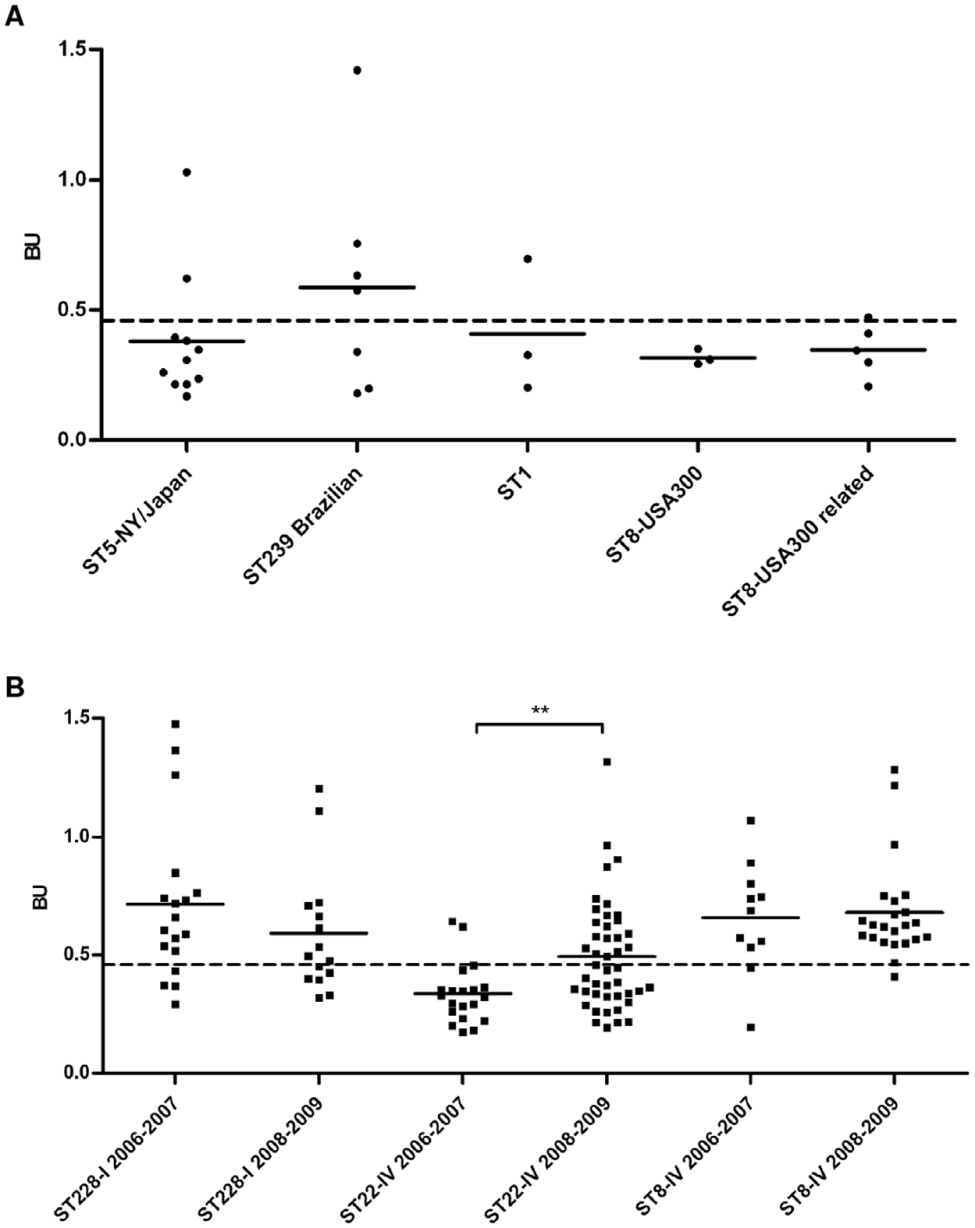
Biofilm production of the main sporadic and epidemic MRSA clones identified in hSR. Panel A: sporadic MRSA clones; panel B: epidemic MRSA clones. The dotted line indicates the threshold separating moderate and strong biofilm producers from non-producers and weak producers with Biofilm Unit (BU) value <0,46. Each symbol represents a single strain. For each clone the average BU value is indicated by the black bar. Three to eleven MRSA strains for sporadic clones and thirty-three to sixty-five MRSA strains for epidemic clones were tested. All MRSA strains tested originated from clinically significant samples (see Materials and Methods). For each epidemic clone (B) the biofilm production is reported for 2006–2007 and 2008–2009 for comparison purposes. Statistically significant differences are indicated by symbols when present: ** indicates a p value <0,01.

As shown in Figure 1, the sporadic MRSA clones ST5-II, ST1-IV and ST8-IV USA300 were associated with low biofilm production, the exception being ST239-III Brazilian clone that was classified as a moderate biofilm producer. Accordingly, the variants of epidemic clones isolated sporadically were weak producers and their incidence decreased rapidly during the study period (data not shown). On the contrary, the predominant variants of clones ST228-I, ST22-IV and ST8-IV were associated with high biofilm production. Interestingly, the clone ST22-IV significantly increased its biofilm production during the study period (p<0,01, Figure 1). In particular, the two predominant variants of the clone ST22-IV, A and B, presented opposite isolation trends, with variant A increasing from 22,2% in 2006 to 36,5% in 2010 and variant B decreasing from 44,4% to 30,7%, and, while the variant B remained a weak biofilm producer, the variant A significantly increased its biofilm production from weak to moderate (p<0,05, data not shown).

### *In vitro* Competition Experiments

The epidemic clones ST228-I and ST22-IV have different genetic background (respectively CC5 and CC22), SCC*mec* type (I and IV) and resistance phenotype (gentamicin-resistant and susceptible). In order to determine if the epidemiological changes can be ascribed to different growth kinetics and/or competitive behavior, we evaluated their growth rate in pure culture and in co-culture. We selected two pairs of strains belonging to the variants B5 (strains 67, isolated in 2006, and 442, isolated in 2009) and A (strains 54, isolated in 2006, and 459, isolated in 2009) of the clones ST228-I and ST22-IV respectively: during the 5-year period, while the variant B5 exhibited a decreasing isolation trend, the variant A was detected with increasing frequency.

**Figure 2:**
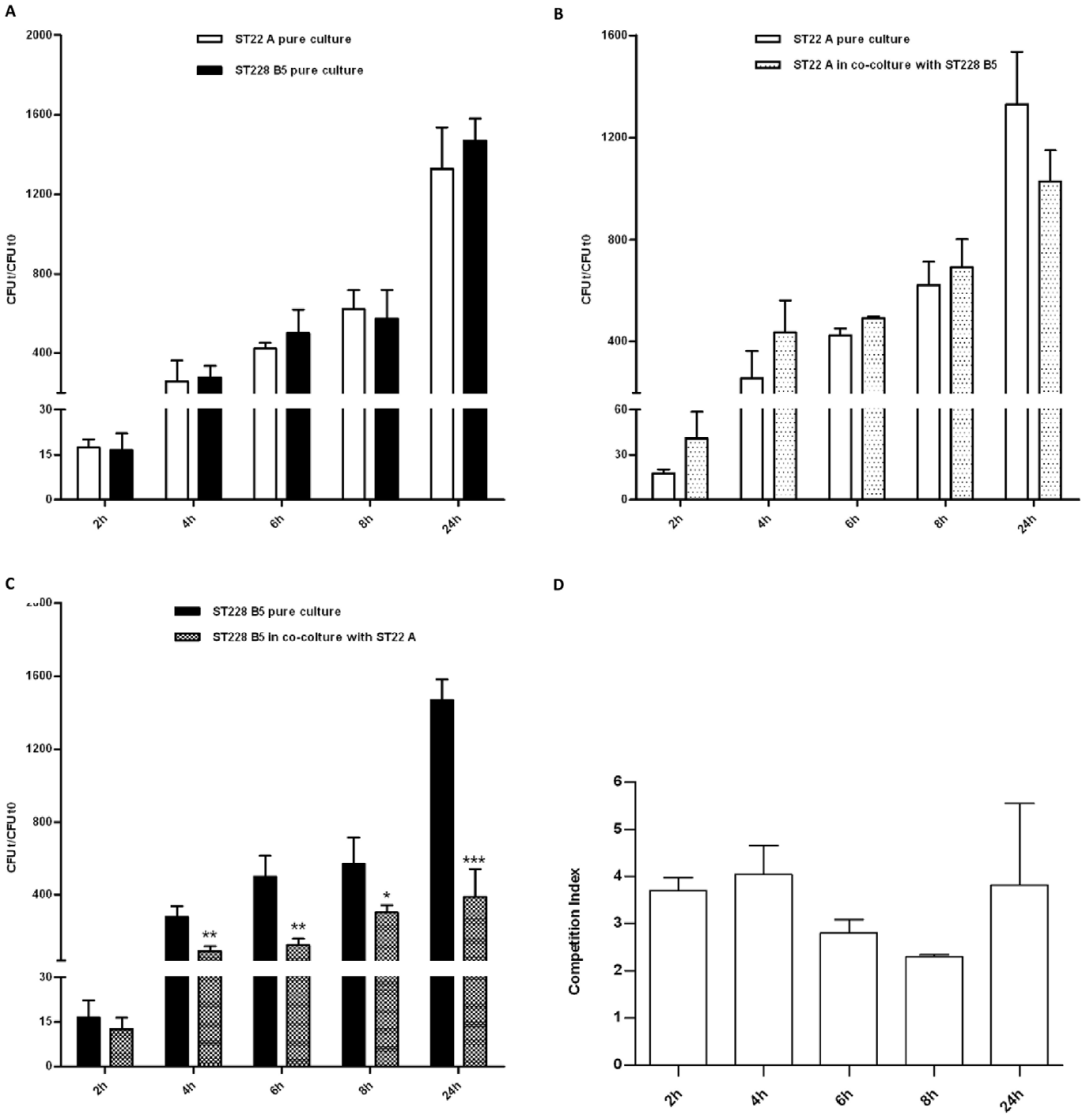
*In vitro* competitive growth cultures. The MRSA clones ST22-IV (variant A, strain 54 isolated from sputum) and ST228-I (variant B5, strain 67 isolated from blood) were grown in pure culture (A) and in co-culture after inoculation in BHI broth at equal ratio (B and C). Growth rate was monitored for 24 hours by colony count after plating and normalizing the output value obtained at each time (t) to the input value at time 0 (t0). CFU: colony forming unit; statistically significant differences are indicated by symbols when present: * indicates a p value <0,05; **: p<0,01; ***: p<0,001. The competition index for mixed culture (D) was calculated at the different time points as the ST22-IV/ST228-I CFU ratio for the output (time t) divided by the corresponding ratio for the input (inoculum at time t = 0).

Figure 2 shows the growth kinetics and the competition indexes (CI) for mixed culture of one pair of strains; similar results were obtained with the second pair of strains. As shown in Figure 2, the two clones presented similar growth kinetics in pure culture; in co-culture, while the variant A of the clone ST22-IV maintained the growth rate observed in pure culture, the growth of the variant B5 of the clone ST228-I was significantly inhibited by the presence of ST22-IV, as confirmed by the CI.

### Invasion in A549 Cell Line Model

To investigate the virulence properties of the clones ST22-IV and ST228-I, we used a well-characterized *in vitro* model, the epithelial cell line A549. In particular, we evaluated the invasion capacity of the major variants of the epidemic clones ST22-IV (variants A and B) and ST228-I (variants B2 and B5) (Table 1).

**Table 1:** Epidemic MRSA clones identified during the study period 2006–2010.

	Frequency of isolation of the clone					
MRSA clone (%)^a^	2006	2007	2008	2009	2010	Major variants of the clone (%)^b^
ST22-IV (33,3%)	20,4%	26,5%	35%	36,7%	38,8%	A (28%); B (25%); C (7%)
ST228-I (26%)	31,8%	33,6%	29,8%	22,7%	20,9%	B2 (16%); B5 (13%); B1 (8%)
ST8-IV (15,8%)	6,8%	16,3%	21,6%	17,2%	17,8%	A1 (23%); A2 (22%); B (21%); C (11%)

a Percent of MRSA strains belonging to each clone, considering only the index cases (n = 499);

b percent of MRSA strains belonging to the major variants of each epidemic clone identified (n = 166 for ST22-IV; n = 131 for ST228-I; n = 80 for ST8-IV).

**Figure 3:**
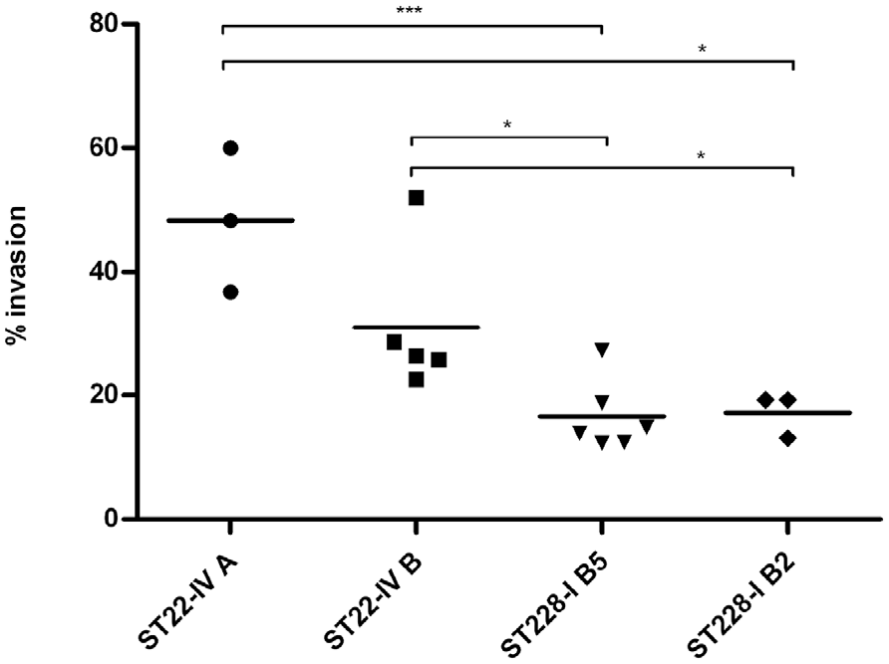
Percentage of invasion of A549 cell line by MRSA clones ST22-IV and ST228-I. Three to six different strains for each predominant variant of the two clones (ST22-IV variants A and B, ST228-I variants B2 and B5) were tested. For ST22-IV strains tested originated from lower respiratory tract (LRT, 37%), pus (37%) and blood (26%); for ST228-I strains tested were isolated from LRT (55%) and blood (45%). The percentage of invasion was determined by plating cellular lysates after 2 hours contact between bacteria and cells’ monolayer, followed by 1 hour treatment with lysostaphin to kill extracellular bacteria. Each symbol represents a single strain; the black bar indicates the average percentage of invasion. Statistically significant differences are indicated by symbols when present: * indicates a p value <0,05; ***: p<0,001.

Both MRSA clones were able to invade the epithelial cells A549 but at different efficiency (Figure 3). The percentages of invasion associated with the variants A and B of the clone ST22-IV (48,3% and 31% respectively) were significantly higher compared to the ones associated with the variants B5 and B2 of the clone ST228-I (16,5% and 17,1% respectively; ST22-IV A vs. ST228-I B5: p<0,001; ST22-IV A vs. ST228-I B2: p<0,05; ST22-IV B vs. ST228-I B5: p<0,05; ST22-IV B vs. ST228-I B2: p<0,05).

### Virulence in a Mouse Model of Acute Lung Infection

To test differences in virulence between the MRSA clones ST22-IV and ST228-I, we used a murine model of acute pneumonia. Variant A of the clone ST22-IV (strain 54) and variant B5 of the clone ST228-I (strain 67) were used to infect C57Bl/6NCrl mice. Lethality was assessed after intratracheal challenge with 5*10^8^ cfu/lung of each strain.

**Figure 4:**
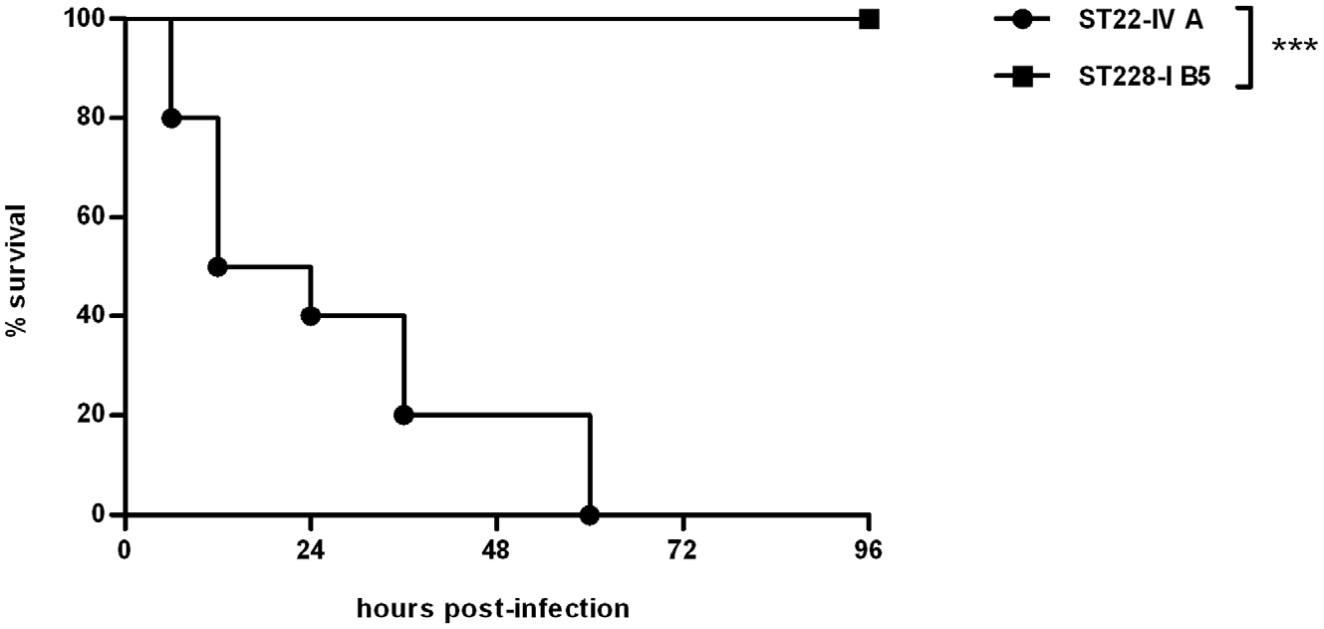
Survival curves of mice infected with MRSA clones ST228-I (variant B5) and ST22-IV (variant A). C57Bl/6NCrl mice were infected intratracheally with 5*10^8^ cfu per mouse of either the variant B5 of the clone ST228-I (strain 67, squares) or the variant A of the clone ST22-IV (strain 54, circles). Two to three independent experiments were pooled (n: 10 mice per MRSA strain). Statistical analysis of pairwise comparison is indicated (Mantel-Cox test): *** indicates a p value <0,001.

As shown in Figure 4, the two MRSA clones determined opposite outcomes: the ST22-IV strain was fully lethal within 60 hours from infection, while the ST228-I strain was attenuated and all mice survived for at least one week after challenge (ST22-IV (variant A) vs. ST228-I (variant B5): p<0,001).

**Figure 5:**
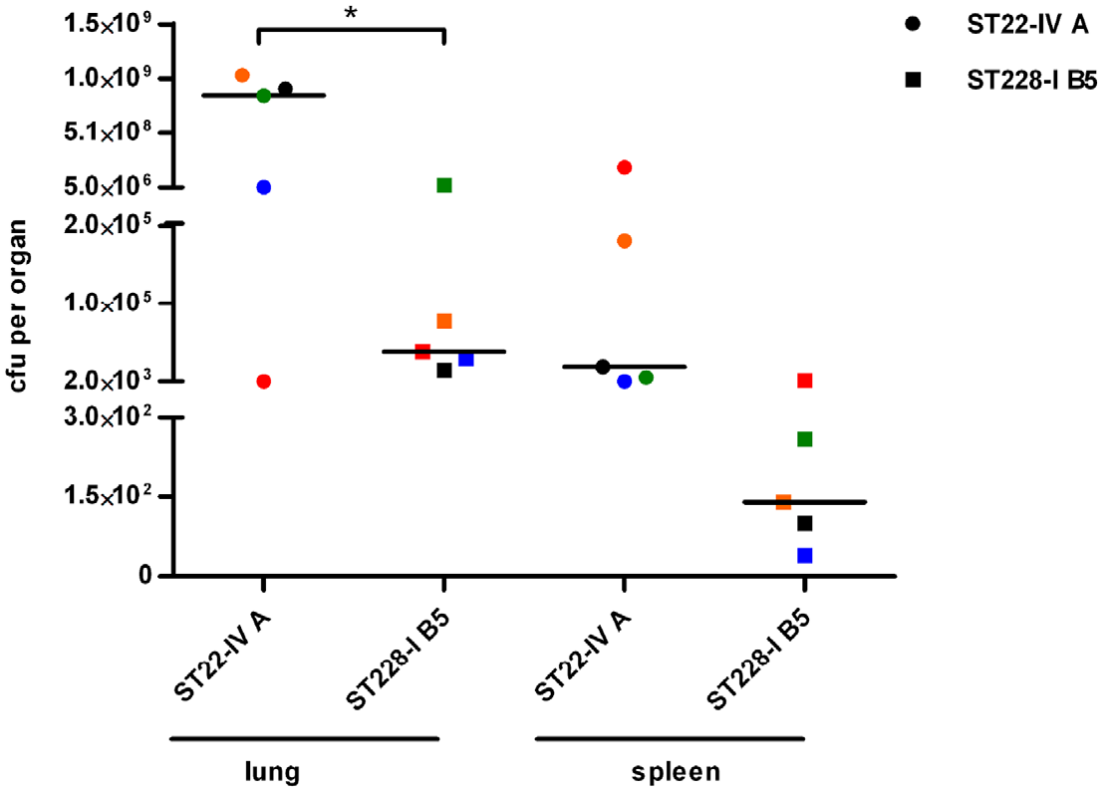
Bacterial load in lung and spleen of mice infected with MRSA clones ST228-I and ST22-IV. C57Bl/6NCrl mice were infected intratracheally with 5*10^8^ cfu per mouse of either the variant B5 of the clone ST228-I (strain 67, squares) or the variant A of the clone ST22-IV (strain 54, circles). Moribund mice infected with ST22-IV clone (n = 5) were sacrificed between 6 and 60 hours from infection; surviving mice infected with ST228-I clone (n = 5) were sacrificed at 72 hours from infection. Each animal is represented by a different color; the black bar indicates the median cfu per organ. Statistically significant differences are indicated by symbols when present: * indicates a p value <0,05.

Bacterial load was then determined in the lungs and spleen of the infected mice (Figure 5). Moribund mice infected with ST22-IV presented a significantly higher bacterial load in the lungs (median value 8,5*10^8^ cfu per lung) compared with mice infected with ST228-I and sacrificed at day 3 (median value 4*10^4^ cfu, p<0,05). Complete clearance of ST228-I strain was observed when the infection was prolonged for 7 days. The bacterial load in the spleen was higher in mice of the ST22-IV group (median 2*10^4^ cfu) and lower in ST228-I group (median 140 cfu), although not statistically significant.

**Figure 6:**
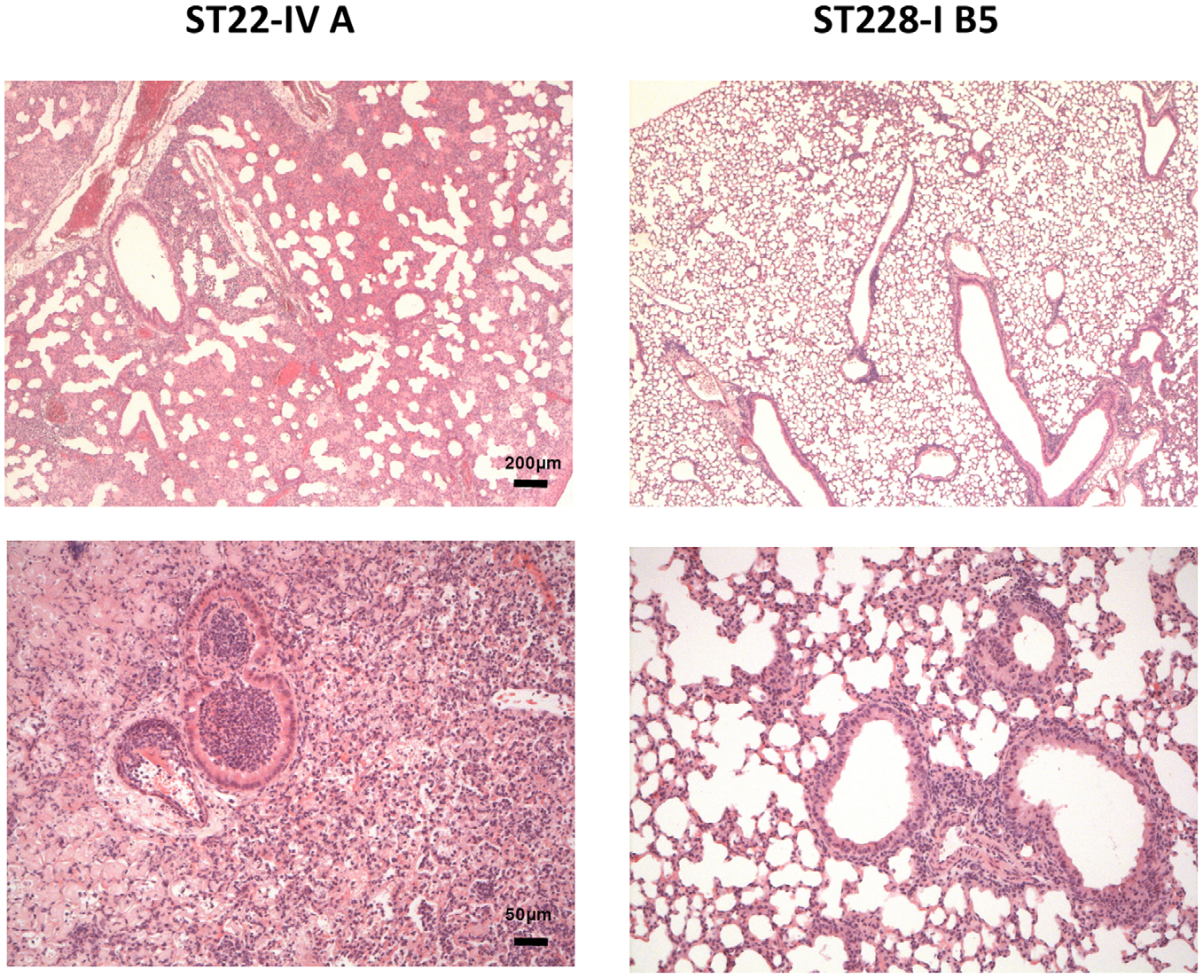
Histopathology in murine lung infected with MRSA clones ST22-IV (variant A) and ST228-I (variant B5). C57Bl/6NCrl were infected intratracheally with 5*10^8^ cfu per mouse of either the variant A of the clone ST22-IV (strain 54) or the variant B5 of the clone ST228-I (strain 67). Mice were sacrificed between 3 and 7 days from infection with ST22-IV (left panel) and ST228-I (right panel), respectively. Lungs were removed en bloc, fixed in 4% paraformaldehyde/PBS and processed for paraffin embedding. Longitudinal sections were stained with hematoxylin and eosin. Upper panel: 2,5× magnification; lower panel: 10× magnification.

To assess clinical strain-specific traits of acute pneumonia, lung histopathology was performed on mice challenged with different MRSA clones (Figure 6). The lungs of mice infected with the ST22-IV clone presented a heavily damaged structure and noteworthy inflammatory infiltrate, while the lungs of mice infected with the ST228-I clone and able to clear the infection presented large areas where the normal pulmonary parenchyma was preserved.

### Genes Coding for Virulence Factors

The MRSA strains used for the competitive growth cultures and for the experiments in the mouse model (strains 54 and 67 belonging to ST22-IV and ST228-I respectively) were characterized for the presence of genes encoding for virulence factors. Both clones shared genes encoding for enterotoxins (*sei, sea, sem, seg, seo*), for factors associated to tissue invasion (*hla, hld, psm-α*), for the clumping factor B (*clfB*), and for the capsular polysaccharide type 5 (*cap5*). The variant B5 of the clone ST228-I also carried genes encoding for other adhesins (*fnbA, efb, ebpS*) and for an additional factor of cellular lysis (*luk E*). The variant A of the clone ST22-IV harbored additional genes encoding for an enterotoxin (*sen*) and for two other toxins (*hlb, hlg*) (Table S1).

### Clinical Data

**Figure 7:**
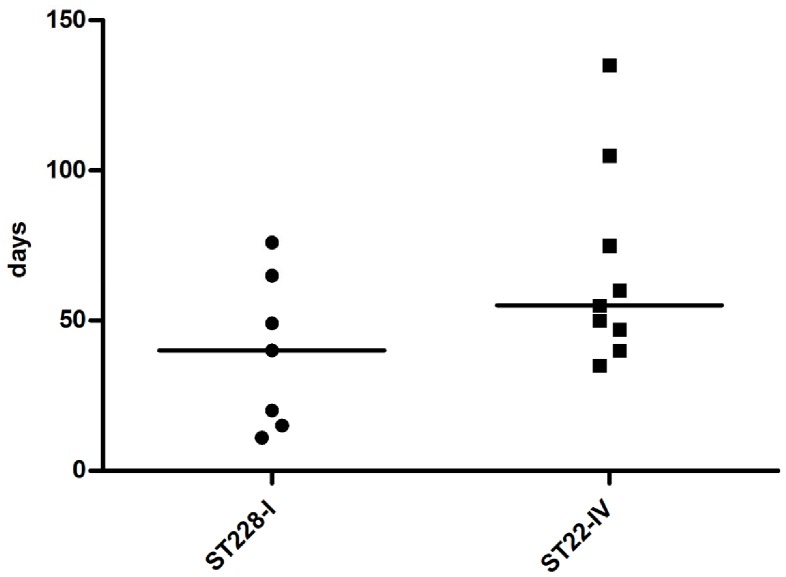
Persistent infections caused by epidemic MRSA clones ST228-I and ST22-IV. Each symbol represents the last MRSA strains isolated from subsequent clinical samples obtained from patients with a previous MRSA isolation. The black bar indicates the median of the days between the first and the last detection of the same MRSA strain.

Between 2009 and 2010 we identified and analyzed 16 cases of persistent infection caused by MRSA strains belonging to one of the predominant variants of the clones ST22-IV and ST228-I. As shown in Figure 7 and Table 2, 9 and 7 patients had a second MRSA isolate belonging to the ST22-IV and to the ST228-I clone respectively. The median of days between the first and the last MRSA detection for the clone ST228-I was 40 days, while for the ST22-IV was 55 days.

**Table 2:** Clinical characteristics of patients with persistent MRSA infection identified between 2009 and 2010.

		Source of MRSA								
Patient	Age (y)^a^	1^st^ sample	2^nd^ sample	3^rd^ sample	4^th^ sample	Days between first and last positive sample	MRSA clone	Underlying disease	Diagnosis	MRSA therapy
370	52	biopsy	BAL^b^	–	–	40	ST22-IV	malignant tumour	MRSA infection	tigecycline
425	76	biopsy	pus (wound)	–	–	47	ST22-IV	–	MRSA decubitus ulcer	–
453	79	blood	blood	pus (abscessus) + blood	–	75	ST22-IV	chronic renal failure	MRSA bacteremia	vancomycin
460	57	BASP^c^	BASP	–	–	35	ST22-IV	benign tumour	MRSA infection	teicoplanin
527	55	blood	blood	–	–	60	ST22-IV	–	MRSA bacteremia	vancomycin
537	64	blood	blood	pericardial fluid	blood	105	ST22-IV	–	MRSA bacteremia	cubicin
575	65	pus (wound)	biopsy	–	–	50	ST22-IV	peripheral arteriopathy	MRSA ulceration	levofloxacin
614	28	vaginal swab	BASP	BAL	–	135	ST22-IV	paraplegia	MRSA infection	levofloxacin + cotrimoxazole
664	62	blood	blood	blood	–	55	ST22-IV	–	MRSA bacteremia	cotrimoxazole
672	82	BAL	sputum	–	–	20	ST228-I	lung cancer	MRSA pneumonia	vancomycin
539	59	sputum	BAL	–	–	65	ST228-I	–	MRSA infection	linezolid
572	64	BASP	BASP	BASP	BASP	40	ST228-I	obstructive hydrocephalus	MRSA infection	*
574	67	BASP	BAL	BASP	BASP	49	ST228-I	benign tumour	MRSA infection	vancomycin
245	70	blood	blood	–	–	15	ST228-I	chronic renal failure + acute myeloid leukemia	MRSA bacteremia	teicoplanin
441	41	BASP	BASP	BASP	blood	76	ST228-I	–	MRSA infection	vancomycin
495	41	BASP	BASP	–	–	11	ST228-I	malignant tumour	MRSA infection	vancomycin

a years;

b broncho-alveolar lavage;

c bronchial aspirate;

* patient transferred to another hospital; - not present.

The average age of patients with persistent infection caused by the clone ST228-I and ST22-IV was 60,5 years and 59,7 years respectively. Cases of resolving MRSA bacteremia, defined as an initial blood culture that yielded MRSA and subsequent blood cultures negative for MRSA after antibiotic treatment, were equally caused by both ST22-IV and ST228-I clones (data not shown). On the contrary, cases of persistent MRSA bacteremia were significantly associated to ST22-IV clone (ST22-IV vs. ST228-I: p<0,001): four out of nine patients (44,4%) infected by the ST22-IV clone had persistent bacteremia, whose median length was 67,5 days, while only one (14,2%) of the seven patients infected by the ST228-I clone had persistent bacteremia that lasted 15 days (Table 2). ST228-I clone in the remaining 85,8% of the cases caused infections of the respiratory tract.

## Discussion

The mechanisms and factors governing the epidemiological dynamics and determining the establishment and successful spreading of a HA-MRSA clone in hospital settings are still unclear. Until the end of the 1990s, the international scenario was dominated by multidrug resistant (MDR) epidemic MRSA clones, suggesting a central role for multidrug resistance in the selection of successful MRSA clones, especially in the hospital environment where the selective antibiotic pressure is notoriously high. Since 2000, however, important epidemiological changes have occurred and classical MDR MRSA clones have been replaced by more susceptible clones. In Europe, these changes have been related to the gentamicin-susceptible ST22-IV clone, otherwise known as EMRSA-15, which has shown a particular ability to substitute other predominant clones in several countries, such as France, United Kingdom, Portugal, Spain, Hungary and Germany [16]-[19], [21].

In line with the epidemiological changes observed in Europe in the last decade, in our institution during the five-year period 2006–2010, we detected the establishment of the ST22-IV clone that displaced the predominant Italian clone ST228-I. We investigated both *in vitro* and *in vivo* virulence properties associated with this phenomenon of clone replacement in hospital settings.

We found a correlation between the successful establishment and persistence in nosocomial settings of MRSA clones and their capacity to produce biofilm, so far demonstrated only for the epidemic Brazilian clone [22]. We also detected the ability of the predominant variant A of the clone ST22-IV to favorably modify its phenotype, thus acquiring a selective advantage over the variant B, a weak biofilm producer associated with a decreasing isolation trend over time. Biofilm constitutes a protective mode of growth that confers to bacteria resistance to antibiotic therapy and host immune response, thus probably increasing their persistence in the host, as well as in the environment and inanimate surfaces, and their pathogenic potential [23].

The role played by genetic background in establishing the success of a specific MRSA clone is unclear. Laurent *et al.* described a correlation between gentamicin susceptibility and the increased capacity of MRSA clones to replicate, establish and spread in nosocomial environments, compared with gentamicin-resistant clones [19]. Both ST22-IV and ST228-I clones presented similar *in vitro* growth kinetics when grown in pure culture, but different behavior in competitive culture as well as different virulence properties. In co-culture, the clone ST22-IV maintained an unaltered growth rate and was able to significantly inhibit the growth of the competitor clone ST228-I.

MRSA infections are often persistent and associated with a slow response to antibiotic therapy as well as with recurrences, and a consequent extended duration of antimicrobial therapy and hospital stay. *S. aureus* has classically been considered as an extracellular pathogen. Conversely, there is growing evidence suggesting the ability of *S. aureus* to internalize and survive in different cell types [24]. Intracellular presence of bacteria may explain the poor response to antibiotics and the development of chronic infections [25]. We noticed a significantly higher capacity of the clone ST22-IV to invade the human alveolar epithelial cells A549 compared to the clone ST228-I, that could enable this clone to elude both the host immune response and the effects of antimicrobial agents, and to cause persistent or recurrent infections. The analysis of sequential MRSA strains, isolated from patients with persistent MRSA infection, identified 16 patients with multiple isolates genotypically indistinguishable from the first (same pulsotype, spa-type and sequence type) and belonging to one of the predominant variants of the clones ST22-IV and ST228-I. ST22-IV, compared with ST228-I, was able to cause a significantly higher percentage of persistent, long lasting bacteremia (44,4% vs. 14,2%, respectively), despite the presence of antimicrobial therapy. On the contrary, ST228-I clone in 85,8% of the cases determined infections of the respiratory tract. Further *in vitro* and *in vivo* studies that aim to evaluate the ability of these clones to persist intracellularly could confirm this data.

Considering that pneumonia caused by MRSA accounts for 20–40% of nosocomial infections and it is still one of the leading causes of death during flu epidemics [26], and considering that more than 35% of our MRSA strains have been isolated from clinically significant respiratory samples obtained from infected patients, we further evaluated the *in vivo* virulence associated with the MRSA clones ST22-IV and ST228-I using a murine model of acute lung infection. Mice challenged with either the ST22-IV or the ST228-I clone showed opposite clinical outcomes. The clone ST22-IV presented a higher virulence compared to clone ST228-I: it seriously damaged the lung, compromising its structure and causing severe inflammation, thus entering the bloodstream and determining sepsis and death in all the mice within 60 hours. This data correlated with the noteworthy *in vitro* invasion capacity associated with this clone. On the contrary, the reduced virulence of the clone ST228-I was associated with the survival of all animals and the clearance of the infection.

The analysis of the pathotypes associated with the two clones revealed that, while ST228-I presented a major number of genes encoding for adhesins (*fnbA, efb, ebpS*), ST22-IV presented a major number of toxins (*hlb, hlg*), whose role is still unclear, although they probably contribute to its higher capacity to invade observed both *in vitro* and *in vivo*. Giese *et al.* have recently shown synergistic activity between the δ-toxin (*hld*) and the β-toxin (*hlb*) that allows *S. aureus* to escape from the phago-endosomes of human epithelial and endothelial cells, thus avoiding degradation [27]. It is not clear if the escape from the phago-endosome leads to the subsequent host cell’s death or if it allows bacteria to survive in the cytosol.

The data reported here suggest that the EMRSA clone ST22-IV could have exploited its capacity to i) increase its biofilm production over time, ii) maintain its growth kinetics in the presence of a competitor clone and iii) be particularly invasive and virulent both *in vitro* and *in vivo*, so as to replace other well-established EMRSA clones and become the predominant European clone. Furthermore, the ability of the clone ST22-IV to determine a higher number of persistent, long lasting bacteremia compared to ST228-I, revealed an additional factor that could help to explain its epidemiological success. In fact, although MDR clones have been progressively replaced by more susceptible clones, the treatment of MRSA infections is increasingly problematic: growing evidence suggests the ability of this pathogen to survive intracellularly, even for prolonged time periods, in the cytoplasm of both phagocytic and non-phagocytic cells [24], [28]. It is presumable that intracellular persistence is a bacterial strategy to subvert immunological defense mechanisms, as well as extracellular bactericidal concentrations of antibiotics. An intracellular niche might then serve as a reservoir for chronic or relapsing infections and/or contribute to chronic carriage of the pathogen and to its spreading in both hospital settings and the community. Further studies using both *in vitro* and *in vivo* models of persistent infection could help to elucidate if these mechanisms of adaptation and persistence are also present in clinical EMRSA clones that we characterized for acute virulence.

## Materials and Methods

### Ethics Statement

Animal studies were conducted according to protocols approved by the San Raffaele Scientific Institute (Milan, Italy) Institutional Animal Care and Use Committee (IACUC) and adhered strictly to the Italian Ministry of Health guidelines for the use and care of experimental animals.

### Bacterial Strains

A total of 575 MRSA non-duplicated strains (499 index cases and 76 secondary cases due to nosocomial transmission), isolated from 2006 to 2010 from hospitalized patients with severe MRSA infection, were included in the study. The strains were isolated from clinical samples originating from lower respiratory tract (LRT, 36,6%), blood (27%), pus (15,8%), biopsy (9,1%) and other sites (11,5%). All these strains were characterized by pulsed-field gel electrophoresis and SCC*mec* typing, which allowed identifying epidemic and sporadic MRSA clones. Epidemic clones were constantly detected during the whole study period and their frequency of isolation was >5% (Table 1); on the contrary, sporadic clones were rarely detected with a frequency of isolation <5%. Each epidemic clone presented major and minor variants (or sub-clones) associated to different pulsotypes that were detected at a frequency >5% and <5% respectively.

### DNA Extraction and PCR for Virulence Factors Genes

Bacterial DNA was prepared from isolated colonies suspended in 100 µl Triton X-100 lysis buffer with 1% Triton and 50 µg/ml lysostaphin, incubated at 37°C for 15 min, heated at 95°C for 15 min. After centrifugation, 5 µl of the supernatant was used for PCR. Selected virulence factors were detected by PCR using primers and protocol described by Diep BA *et al.* [29].

### Biofilm Production

Quantitative determination of biofilm production was performed in a microtiter plate under static conditions following the procedure previously described by Stepanovic S *et al.* [30], and using SH1000 as positive control and TSB as negative control. The biofilm units (BU) were calculated using the formula: OD_biofilm_/OD_growth_. Classification: BU≤0.230 non-producers, BU>0.230 and ≤0.460 weak producers, BU>0.460 and ≤0.920 moderate producers, BU>0.920 strong producers. Each MRSA strain was tested in triplicate and each experiment was repeated three times independently. For ST228-I clone the isolates tested belonged to major variants B1, B2 and B5 and originated from blood (64%), LTR (30%) and pus (6%) (Table 1). For ST22-IV and ST8-IV clones isolates tested belonged to variants A, B, C and A1, A2, B, C respectively and originated from blood (42%), LRT (32%), pus (13%) and biopsy (13%). For ST5-NY/Japan clone strains originated from LRT (45%), blood (20%), pus (17,5%) and biopsy (17,5%); for ST239-Brazilian clone strains originated from blood (42%), LRT (42%) and pus (16%); for ST1 clone isolates originated from pus (66,6%) and LRT (33,3%); ST8-USA300 and ST8-USA300-related, being community-acquired MRSA clones, caused mainly skin and soft tissue infections (SSTI) and 62% of isolates tested for biofilm production originated from pus, 25% from LRT and 13% from blood. Figure 1 reports the average BU value for each MRSA strain tested.

### Competitive Growth Experiments

Two MRSA isolates belonging to each of the ST22-IV and ST228-I clones were tested against each other in both pure and mixed cultures for competitive growth. Each pair of strains were inoculated at equal ratio (1 OD_600_, optical density) from mid-exponential phase pure cultures in a final volume of 30 ml of Tryptic Soy Broth and incubated at 37°C with agitation (180 rpm) for 24 hours. Growth rates of pure and mixed cultures were monitored every 2 hours up until 8 hours and at 24 hours, by dilution plating on Tryptic Soy agar (TSA) plates with or without gentamicin (32 µg/mL), to discriminate the two MRSA clones, and CFU count. Each experiment was repeated three times independently. The competition index (CI) for mixed culture was calculated as the ST22-IV/ST228-I CFU ratio for the output (obtained at the different time points) divided by the corresponding ratio for the input (inoculum at time t = 0) [31].

### Invasion Assay

Bacterial invasion was investigated using the A549 cell line as previously described by Liang X *et al* [32]. A549 cell line (ATCC number CCL-185) was obtained from LGC Standards. Briefly, 2×10^5^ cells were seeded on 24-well flat-bottom plates and incubated for 2 hours at 37°C with 1–2×10^6^ bacteria from the mid-exponential phase; adherent and extracellular bacteria were killed by lysostaphin treatment (20µg/ml) for 1 hour at 37°C; after washing wells and treating with Trypsin-EDTA, followed by Triton X-100 0,025% to detach and lyse cells, internalized bacteria were serially diluted and plated on TSA for counting. Each MRSA strain was tested in duplicate and each experiment was repeated three times independently. Figure 3 reports the average percentage of invasion for each MRSA strain tested.

### Mouse Model of Acute Lung Infection

C57Bl/6NCrl mice (20–22 gr) were purchased by Charles River. Mice were housed in filtered cages under specific-pathogen conditions and permitted unlimited access to food and water. Prior to animal experiments, the clinical MRSA strains were grown for 3 h to reach exponential phase. Next, the bacteria were pelleted by centrifugation (2700 g, 15 min), washed twice with sterile PBS and the OD of the bacterial suspension was adjusted by spectrophotometry at 600 nm. The intended number of cfu was extrapolated from a standard growth curve [33], [34]. Appropriate dilutions with sterile PBS were made to prepare the inoculum of 5×10^8^ cfu per mouse. Mice were anesthetized with 375 mg/kg Avertin (2,2,2-tribromoethanol, 97%; Aldrich), the trachea directly visualized by a ventral midline incision, exposed and intubated with a sterile, flexible 22-g cannula attached to a 1 ml syringe according to established procedures [35], [36]. Mice were monitored for mortality and sacrificed by CO_2_ administration up to 7 days. To determine the bacterial load murine lungs and spleens were aseptically excised, homogenized, and plated onto TSA agar with or without gentamicin (32 µg/mL).

### Histological Examination

Moribund mice were sacrificed by CO_2_ administration while surviving mice were sacrificed after 7 days from infection, lungs were removed en bloc and fixed in 4% paraformaldehyde/PBS, at 4°C for 24 h, and processed for paraffin embedding. Longitudinal 2-µm sections taken at regular intervals were obtained using a microtome from the proximal, medial and distal lung regions. Sections were stained with Haematoxylin-Eosin according to standard procedures and examined blindly.

### Statistical Analysis

Data were analyzed with the software GraphPad Prism 5. Data concerning biofilm production, competitive cultures and invasion were analyzed using two-tailed non-parametric Student’s t test. For *in vivo* experiments survival data were analyzed with the Log Rank test (Mantel-Cox). Value of *p*<0,05 was considered to be statistically significant.

## Supporting Information

Table S1 Virulence characteristics of the MRSA strains used for in vivo experiments.
(DOC)
