# Supplementary material for: Factors Contributing to Epidemic MRSA Clones Replacement in a Hospital Setting
Source: PLoS One. 2012 Aug 14;7(8):e43153. doi: 10.1371/journal.pone.0043153 (PMC3419217; doi:10.1371/journal.pone.0043153)
Supplement: Table S1 — Virulence characteristics of the MRSA strains used for in vivo experiments. (DOC) [file pone.0043153.s001.doc]

**Table S1. Virulence characteristics of the MRSA strains used for *in vivo* experiments.**

| **Virulence characteristic** | **ST22-IV variant A strain 54** | **ST228-I variant B5 strain 67** |
| --- | --- | --- |
| BU/classification | 0,47/moderate | 0,70/moderate |
| BU of the clone/classification | 0,51/moderate | 0,88/moderate |
| % of invasion | 48,3% | 27,1% |
| % of invasion of the clone | 48,3% | 16,5% |
| *sea* (staphylococcal enterotoxin A) | + | + |
| *seb* (staphylococcal enterotoxin B) | - | - |
| *sec* (staphylococcal enterotoxin C) | - | - |
| *seg* (staphylococcal enterotoxin G) | + | + |
| *seh* (staphylococcal enterotoxin H) | - | - |
| *sei* (staphylococcal enterotoxin I) | + | + |
| *sek* (staphylococcal enterotoxin K) | - | - |
| *sel* (staphylococcal enterotoxin L) | - | - |
| *sem* (staphylococcal enterotoxin M) | + | + |
| *sen* (staphylococcal enterotoxin N) | + | - |
| *seo* (staphylococcal enterotoxin O) | + | + |
| *seq* (staphylococcal enterotoxin Q) | - | - |
| *tst* (toxic shock syndrome toxin) | - | - |
| *pvl* (Panton-Valentine leukocidin) | - | - |
| *lukE* (leukotoxin E) | - | + |
| *psmα* (phenol soluble modulin α) | + | + |
| *hla* (α-hemolysin) | + | + |
| *hlb* (β-hemolysin) | + | - |
| *hld* (δ-hemolysin) | + | + |
| *hlg* (γ-hemolysin) | + | - |
| *clfA* (clumping factor A) | - | - |
| *clfB* (clumping factor B) | + | + |
| *cap5* (capsular polysaccharide type 5) | + | + |
| *cap8* (capsular polysaccharide type 8) | - | - |
| *fnbA* (fibronectin binding protein A) | - | + |
| *efb* (extracellular fibrinogen binding protein) | - | + |
| *ebpS* (elastin binding protein) | - | + |

BU: biofilm units; classification: BU≤0.230 non-producers, BU>0.230 and ≤0.460 weak producers, BU>0.460 and ≤0.920 moderate producers, BU>0.920 strong producers.
